# Supplementary material for: Poly(alkyl-biphenyl pyridinium)-Based Anion Exchange Membranes with Alkyl Side Chains Enable High Anion Permselectivity and Monovalent Ion Flux
Source: Membranes (Basel). 2023 Feb 3;13(2):188. doi: 10.3390/membranes13020188 (PMC9967815; doi:10.3390/membranes13020188)
Supplement: Supplementary file 1 [file membranes-13-00188-s001.zip › membranes-2141690-supplementary.pdf]

Supporting Information

Poly(alkyl-biphenyl pyridinium)-based anion exchange membranes with alkyl side chains enable high anion permselectivity and monovalent ion flux

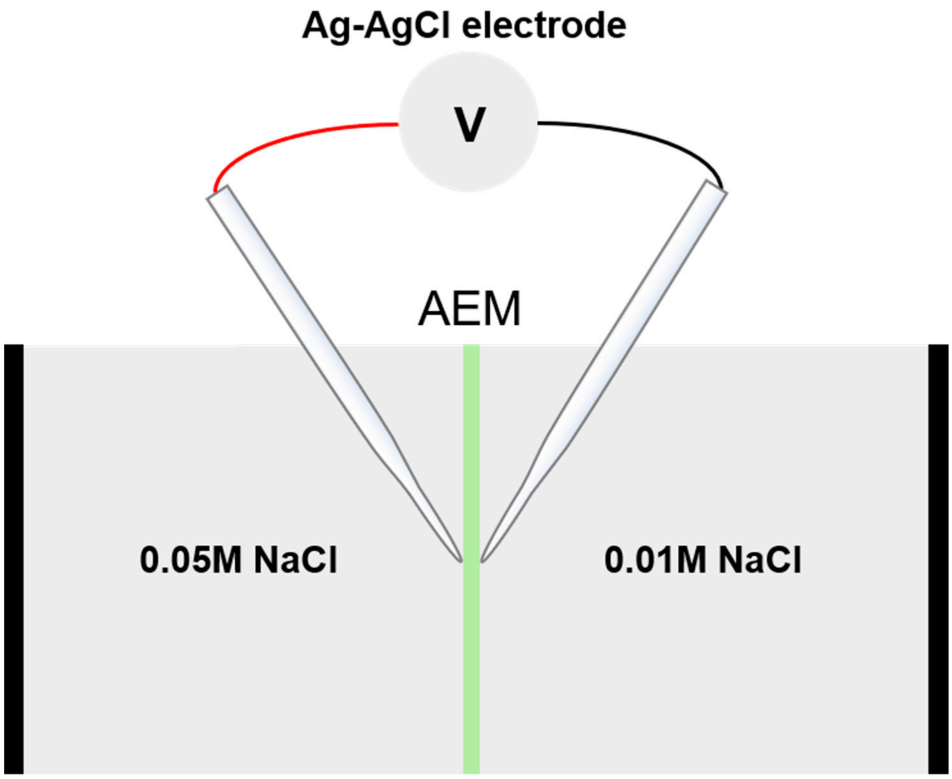

Figure S1. Schematic illustration for Membrane transport number.

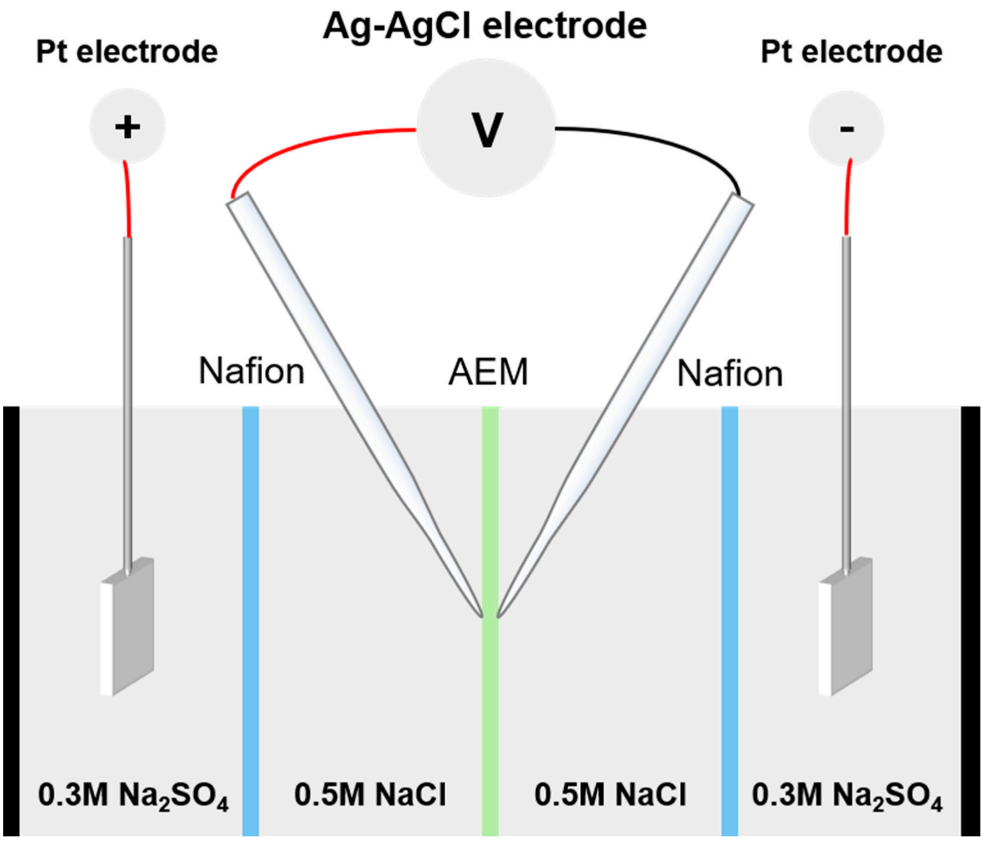

Figure S2. Schematic illustration for current-voltage (I-V) curves and membrane area resistance.

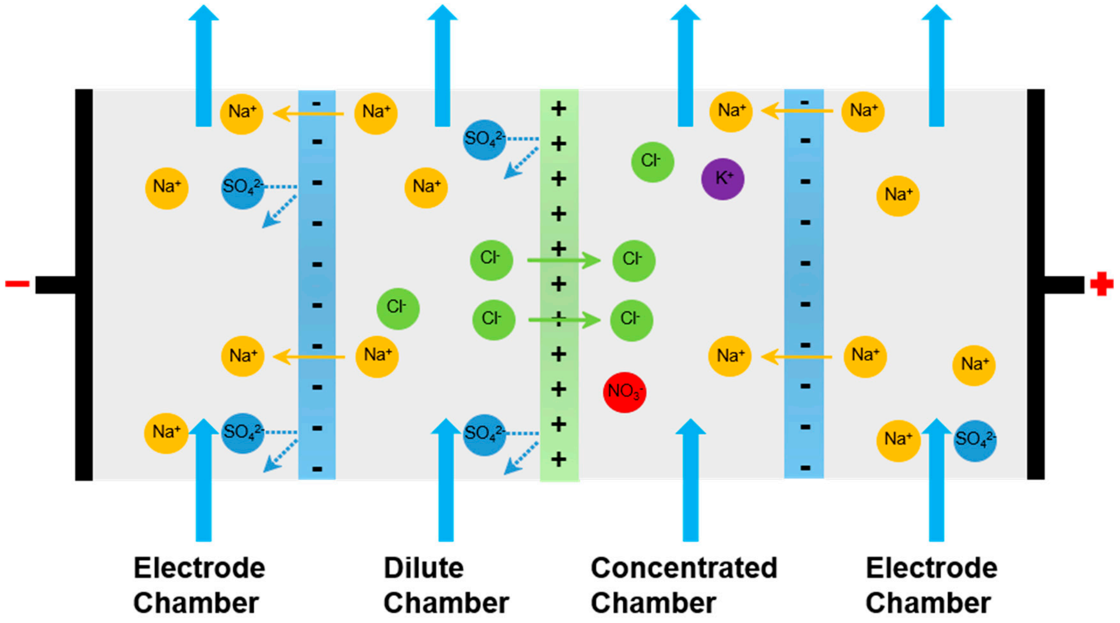

Figure S3. Schematic illustration for the ED process.

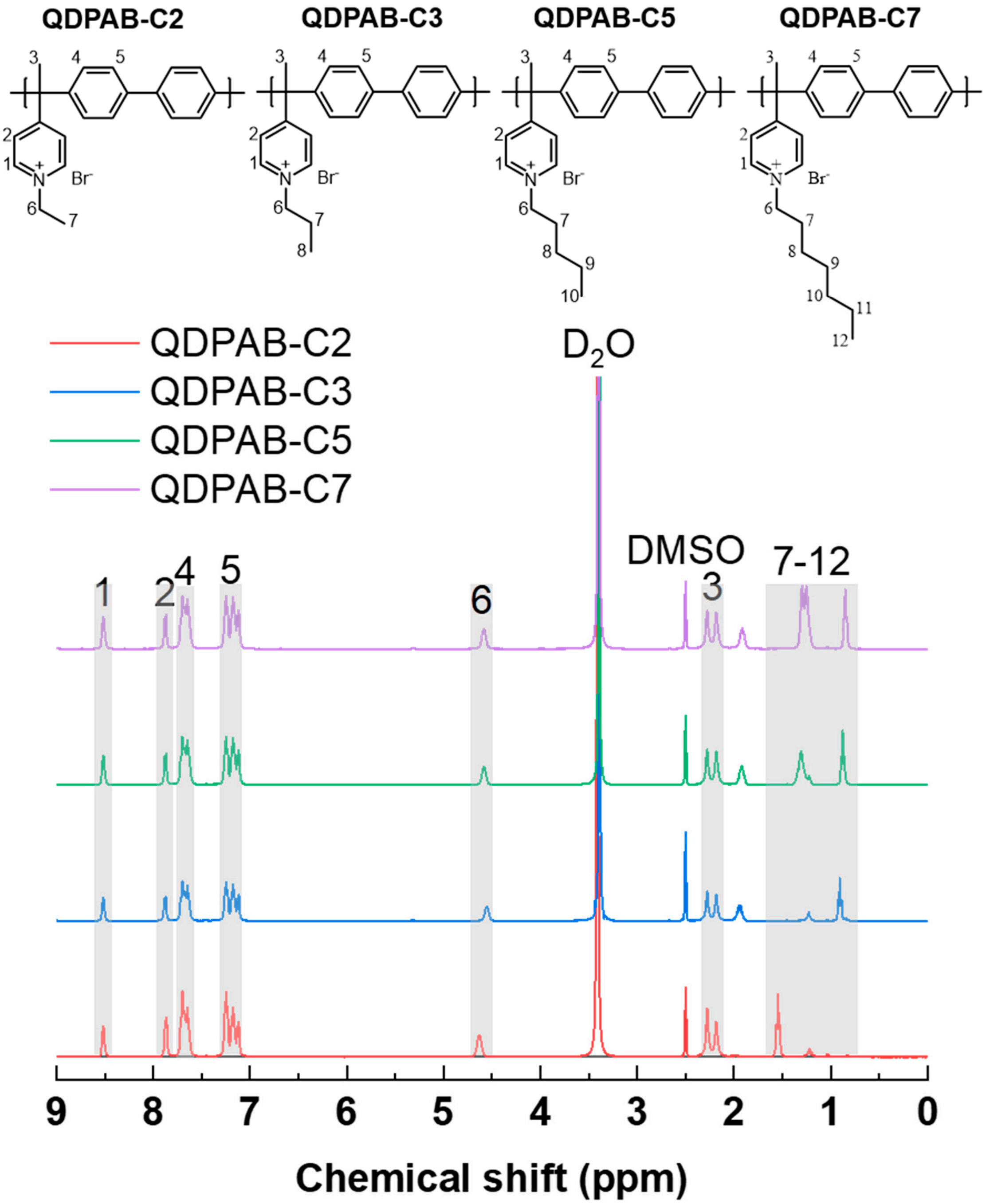

Figure S4. ^{1}H NMR spectra of QDPAB-C2, QDPAB-C3, QDPAB-C5 and QDPAB-C7 membranes.
